# Prevalence of *Mycobacterium tuberculosis* infection as measured by the QuantiFERON-TB Gold assay and ESAT-6 free IGRA among adolescents in Mwanza, Tanzania

**DOI:** 10.1371/journal.pone.0252808

**Published:** 2021-06-07

**Authors:** Kidola Jeremiah, Eric Lyimo, Christian Ritz, George PrayGod, Kathryn Tucker Rutkowski, Karen Smith Korsholm, Morten Ruhwald, Dereck Tait, Harleen M. S. Grewal, Daniel Faurholt-Jepsen

**Affiliations:** 1 Mwanza Research Centre, National Institute for Medical Research, Mwanza, Tanzania; 2 Department of Nutrition, Exercise and Sports, University of Copenhagen, Copenhagen, Denmark; 3 International AIDS Vaccine Initiative (IAVI,), New York City, New York, United States of America; 4 Department of Infectious Immunology, Centre for Vaccine Research, Statens Serum Institut (SSI), Copenhagen, Denmark; 5 Department of Vaccine Development, Centre for Vaccine Research, Statens Serum Institut (SSI), Copenhagen, Denmark; 6 Foundation of Innovative New Diagnostics, Geneva, Switzerland; 7 International AIDS Vaccine Initiative (IAVI) NPC, Cape Town, South Africa; 8 Department of Clinical Science, BIDS Group, Faculty of Medicine, University of Bergen, Bergen, Norway; 9 Department of Microbiology, Haukeland University Hospital, Bergen, Norway; 10 Department of Infectious Diseases, Rigshospitalet, Copenhagen, Denmark; The University of Georgia, UNITED STATES

## Abstract

**Background:**

The prevalence of latent tuberculosis infection (LTBI) is vastly higher than that of tuberculosis (TB) disease and this enormous reservoir of individuals with LTBI impacts the global TB control strategy. Adolescents are at greatest risk of TB infection and are thus an ideal target population for a potential effective TB vaccine to be added to the current BCG programme as it could reduce the number of latent infections and consequently the number of adults with TB disease. However, LTBI rates are often unknown for this population. This study aims to estimate the magnitude of LTBI and to determine if Tanzanian adolescents would be a good population for a prevention of TB infection trial.

**Methods:**

This was a descriptive cross-sectional study that recruited 193 adolescents aged 12 and 16 years from government schools and directly from the community in Mwanza Region, Tanzania. Socio-demographic characteristics were collected for all enrolled participants. Blood was drawn and tested using QuantiFERON-TB Gold In-Tube (QFT-GIT), and Early Secretory Antigenic Target-6–Free Interferon-gamma Release Assay (ESAT-6 free IGRA). Concordance between QFT-GIT and ESAT-6 free IGRA was evaluated using the McNemar’s test.

**Results:**

Overall estimates of LTBI prevalence were 19.2% [95%CI, 14.1; 25.2] and 18.6% [95%CI, 13.6; 24.6] as measured by QFT-GIT IGRA and ESAT-6 free IGRA, respectively. The 16-year-old cohort had a higher LTBI prevalence (23.7% [95%CI, 16.1; 32.9]) as compared to 12-year-old cohort (14.6% [95%CI, 8.6; 22.7]) as measured by QFT-GIT IGRA. When measured by ESAT-6 Free IGRA, LTBI prevalence was 24.7% (95%CI, 16.9; 34.0) for the 16-year-old cohort and 12.5% (95%CI, 7.0; 20.3) among the 12-year-old cohort. According to both tests the prevalence of TB infection and the corresponding annual risk of tuberculosis infection (ARTI) and force of infection were high and increased with age. Of all enrolled participants, 97.4% had concordant results for QFT-GIT IGRA and ESAT-6 free IGRA (p = 0.65).

**Conclusions:**

The prevalence of LTBI and the associated ARTI and force of infection among adolescents is high and increases with age in Mwanza Region. There was a high concordance between the QFT-GIT and the novel ESAT-6 free IGRA assays. These findings suggest Mwanza is a promising area to conduct novel TB vaccine research prevention of infection (POI) studies targeting adolescents.

## Introduction

*Mycobacterium tuberculosis* (*M*.*tb*.) is the main agent of tuberculosis (TB); the bacterium is airborne and transmittable between individuals with TB disease, it is even transmittable during the initial period of treatment. In 2019 the World Health Organization (WHO) reported 10 million TB cases, of whom 12% (<15 years) were children, and 1.2 million deaths due to TB, with most TB deaths occurring in low- and middle-income countries [1]. Approximately 1.7 billion people are infected with *M*.*tb*. [1] and hence at risk of developing active TB disease. Although the burden of TB disease was falling globally, that is, in all WHO regions and in most countries [1], COVID -19 pandemic has pushed back the global tuberculosis control progress resulting by 20% drop in diagnosis and treatment worldwide [2]. The risk of *M*.*tb*. infection remains much higher and is greater than the risk of developing active TB disease, with an estimated 5%–15% lifetime risk of progression from latent TB infection (LTBI) to TB disease. The largest TB risk is among children, newly infected individuals, those with a compromised immune system (e.g. due to HIV, malnutrition, diabetes, or ageing) [3], and among those exposed to immunomodulating treatment [4].

An improved and safer preventive TB vaccine is urgently needed to combat the disease alongside current tools for TB prevention, diagnostics, and treatment. The 100-year-old Bacille Calmette-Guérin (BCG) vaccine, unfortunately, seems to have had a minimal effect on global TB incidence despite its widespread use [5]. While BCG is effective in preventing severe forms of TB in infants and young children it has shown limited protection against pulmonary TB in adolescents and adults, the populations primarily responsible for transmitting the disease [6]. A vaccine targeting adolescents and young adults not yet infected could prevent infection and potentially progression to disease in those infected maximizing TB control [7–9] thereby interrupting transmission.

LTBI incidence rate have been demonstrated to be high among adolescents in South Africa [10–13], and interferon-gamma (IFN-γ) release assays (IGRAs) conversion has been shown to be a clear risk factor for TB disease in this age group [14].

Adolescents are therefore a suitable population for prevention-of-infection (POI) trials [15, 16]. In Mwanza, Tanzania, a region of interest for TB vaccine studies the LTBI rates are largely unknown for this target population despite the region reporting a high number of cases of TB disease yearly [17]. Although previously reported LTBI prevalence data among adults in the region suggest a 4% annual conversion rate [18], a more accurate LTBI incidence needs to be determined among the adolescents in the same region to appropriately inform both the international and local community to design and power TB vaccine trials with infection endpoints.

LTBI is defined by detection of cell-mediated immune response towards *M*.*tb*. antigens either by *in vivo* in skin tests or *in vitro* using IGRA [19]. Early Secretory Antigenic Target-6 (ESAT-6) is a component of the H56 fusion protein used in the H56: IC31 vaccine [20], a novel TB subunit vaccine, but ESAT-6 is also one of the antigens used to stimulate IFN-γ production in the QuantiFERON-TB Gold In-Tube (QFT-GIT) IGRA assay [21]. Hence, successful vaccination with H56 leads to false-positive QFT which disqualifies ESAT-6-based vaccines for POI trials using QFT to detect *M*.*tb*. infection. To address this issue we have developed an ESAT-6 free IGRA, where ESAT-6 is substituted by fragments of Rv3615c and other *M*.*tb*. specific and highly recognized antigens [21]. The aim of this study was to determine if Tanzanian adolescents would be a good population for POI trial by assessing the prevalence and annual risk of tuberculosis infection (ARTI) of TB determined by QFT assays in two groups of 12-and 16-year-old adolescents in Mwanza, and also to evaluate the ESAT-6 free IGRA.

## Methods

### Study design and setting

This was a cross-sectional descriptive study with no experimental interventions to determine the prevalence of LTBI as well as to estimate the ARTI among 12- and 16-year-old adolescents using QFT-GIT and ESAT-6 free IGRA. It was conducted from June 2016 to June 2017 in the Mwanza region, the region with the second-highest number of TB case notifications in Tanzania [17]. Mwanza Region is located at the southern end of Lake Victoria and divided into seven districts. In 2012, the region had a total population of 2,772,509 (all ages), of which 395,049, (14.2%), were between 12 and 16 years of age [22]. The chosen study population was from the Ilemela and Nyamagana districts which in 2012 reported to have 14.8% (50,751/343,001) and 14.1% (51,075/363,452) adolescents between 12 and 16 years of age out of the total population for those districts respectively [22].

### Participant eligibility

The study was planned to recruit 96 adolescents who were 12 years of age and 96 adolescents who were 16 years of age, for a total of at least 192 adolescents. Potential participants were enrolled in the study if they had completed the process of written informed consent and assent, were residents of either the Ilemela or Nyamagana district in Mwanza Region, and were 12 or 16 years of age at the enrolment visit. Excluded were those who were HIV-infected (self-report), had an axillary temperature of >38°C, reported chronic illness likely to impair their immune system, or were on immune-modulating medicine (e.g. prednisolone).

### Recruitment of participants

Participants were recruited from schools or directly from the community. Various methods of recruitment were used, for example, through referrals, mobilisation meetings conducted at schools, in TB hot spot areas in the community, and the solicitation of participants previously known to the clinical site. During mobilisation meetings potential participants were educated on the symptoms of TB and the benefits of early diagnosis and treatment. Those interested were requested to register their names and provide their parent’s or guardian’s phone contact. On the next day, participants and their parents or legal guardians were first contacted by the study staff to be informed of the study and educated about TB. Interested participants and their parents or legal guardians were scheduled for appointment to participate in the informed-assent and the informed-consent process. Eligible persons were requested to come to the research clinic at NIMR Mwanza at 8:00 am after an overnight fast of at least 8 hours to receive further study information, for consenting, and for study procedures. The participants were then evaluated for study eligibility once the participant’s assent and the parents’ or legal guardians’ consent were obtained.

### Data collection

Using the REDCap web-based application available at https://www.project-redcap.org, a database was designed and information collected on key demographic characteristics, such as age, gender, education, religion and residential district for all enrolled participants.

### Laboratory procedures

#### Blood specimen collection

Eligible participants were requested to provide a whole blood sample for the laboratory tests. A trained phlebotomist did a blood draw from each participant of approximately 4 mL of whole blood, 1 mL each into three tubes of QFT-GIT and one tube of ESAT-6 free IGRA [21, 23]. Blood was collected in a sequence of four tubes: TB-NIL tube, TB antigen tube, an ESAT-6 free tube and TB mitogen tube. Once the blood had been collected the tubes, were gently inverted 10 times (except TB-NIL tube) and maintained at ambient temperature (18–25°C) at the site clinic before being shipped to a laboratory for processing within 2 hours of blood-taking.

#### QuantiFERON-TB gold in-tube and ESAT-6 free IGRA assays

ESAT-6 free IGRA test tubes comprising lyophilized peptides and heparin mixture in vacutainer tubes drawing 1 ml blood were prepared as described elsewhere [21].

Blood samples were drawn directly into the QFT-GIT vacutainer tubes as per manufacturer instructions and the ESAT-6 free IGRA tube was drawn in sequence after the TB antigen tube and before TB mitogen tube. The vacutainers were immediately mixed, and within 2 hours they were incubated at 37°C for 16 to 20 hours. After the incubation, the tubes were centrifuged at 2000 x*g* for 15 minutes, then the plasma was harvested and stored at –80°C until further testing. The QFT-GIT enzyme-linked immunosorbent assay (ELISA, lot no. 55402033) test for the response to *M*. *tb*. infection was performed following the manufacturer instructions [24]. Only the sample layout format on the ELISA microplate was altered to accommodate the ESAT-6 free IGRA assay plasma; otherwise, the layout followed the QuantiFERON-TB Gold Plus ELISA format.

#### Optical density analysis and conversion

The Beckman Coulter DTX 800 Multimode Detector ELISA reader was used to determine the optical density of the QFT-GIT assay ELISA microplates. The optical density data were obtained and fed to the QuantiFERON-TB Gold Plus version 2.71 Build 08 for analysis. The software converts optical density values to a quantitative IFN-γ *M*.*tb*. response in IU/mL. A positive response, according to the manufacturer, is a value of ≥0.35 IU/mL (*M*.*tb*. antigen or ESAT-6 free IGRA minus Nil) and ≥25% of the Nil plasma value.

### Ethics statement

Ethical permission to conduct the study was granted by the Medical Research Coordinating Committee (MRCC) of the National Institute for Medical Research (NIMR) in Tanzania with number NIMR/HQ/R.8a/Vol.IX/2318 and by the Lake Zone Institutional Review Board (LZIRB). Only participants giving written informed assent and their parent’s or guardian’s written informed consent were included. Oral and written information in Swahili was provided to all participants prior to obtaining informed oral and written assent and consent. Written informed assent and consent were obtained to all participants before conducting any study related activities. A copy of the signed assent and consent forms was given to the participants and their parents or legal guardians for their records.

### Data management and statistics

Completed source documents were transcribed into the study database and verified by independent double entry. Statistical analysis was performed using SAS 9.4 (SAS Institute, Cary, NC). LTBI prevalence among 12- and 16-year-old adolescents, as measured by the QFT-GIT assay and ESAT-6 free IGRA assay, was calculated for all enrolled participants with non-missing IGRA data.

The ARTI among 12- and 16-year-old adolescents, as measured by the QFT-GIT IGRA, was calculated for all enrolled participants with non-missing IGRA data. The ARTI is an age-specific measure of the average risk for *M*. *tb*.-infection over the lifetime of the participant [25]. It was calculated using the formula R = 1 –(1 –P)^1/A^, where R is the annual risk of infection (expressed as a fraction), P is *M*.*tb*. prevalence of the age group (expressed as a fraction), and A is the age of the participants. Summaries of ARTI are presented by age and gender. The concordance between QFT-GIT and ESAT-6 free IGRA was evaluated using McNemar’s test. The LTBI prevalence among 13- to 15-year-olds was estimated by assuming a linear trend between the prevalence determined in 12- and 16-year olds as determined by the QFT-GIT and ESAT-6 free IGRA. The ARTI for 13- to 15-year-olds was also estimated by using the estimated LTBI prevalence for each of these age groups as measured by the QFT-GIT and ESAT-6 free IGRA. The force of infection of LTBI is the “proportion of susceptible individuals that have become infected with *M*.*tb*. in a specified period”, calculated using changes in age-specific prevalence rates [25]. The formula used to calculate the force of infection is F = ΔP / [1 –P], where F is the risk of *M*.*tb*. infection (expressed as a fraction) in the residual pool of non-infected individuals for a specific age group, ΔP is the annual change in prevalence (expressed as a fraction) for a specific age group, and P is the *M*.*tb*. prevalence (expressed as a fraction) of the age group.

## Results

A total of 193 adolescent were enrolled from after being determined to be eligible for this study. Of those enrolled, 96 were in the 12 year old cohort (5 participants in the 12 year old cohort were 11 years old), and 97 were in the 16 year old cohort (12 participants in the 16 year old cohort were 15 years old). There was a higher proportion of females than males in the 12 year old cohort as compared to the 16 year old cohort where the proportion of females and males was similar Table 1. About 65% of the participants reported staying with their biological parents. Seven and 9% of the 12 year old and the 16 year cohort, respectively reported exposure to pulmonary TB within 12 months of the time of enrolment. Further demographic data is presented in Table 1.

**Table 1:** Demographic and baseline characteristics of 193 adolescent assessed for latent tuberculosis infection^a^.

Characteristics	12 years old^b^	16 years old^b^
	N = 96	N = 97
**Female gender**	60 (62)	50 (52)
**Participant lives with**		
Parent(s)	62 (65)	64 (66)
Other	34 (35)	33 (34)
**Known pulmonary tuberculosis exposure for the last 12 months**	7 (7)	9 (9)
**Household contact smoking history**		
Yes, currently smoking every day	15 (16)	6(6)
Yes, currently smoking someday	3 (3)	4(4)
Not currently smoking, but did regularly in past	4 (4)	6(6)
Never smoked	74 (77)	81(84)
**Parent/guardian occupation**		
Salaries	7 (7)	7 (7)
Self-employed	74 (77)	73 (75)
Housewife	8 (8)	13 (13)
Student	2 (2)	0
Unemployed	5 (5)	3 (3)
Others	0	1 (1)
**Parent/guardian education level**		
No formal education	13(14)	13 (13)
Incomplete primary	5 (5)	3 (3)
Complete primary	65 (68)	71 (73)
Secondary/ High school	9 (9)	8 (8)
Secondary/tertiary college	4(4)	2 (3)
**Number of adult household member**, mean (SD)	3.0 (1.30)	3.3 (1.45)
**Number of child household member (<18 years)** mean (SD)	4.1 (1.78)	3.6 (1.73)
**Number of rooms in the house mean (SD)**	4.3 (2.48)	4.1 (1.55)
**Primary cooking area**		
Separate room/ kitchen	15 (16)	29 (30)
Main room	14 (15)	8 (8)
Separate building	34 (35)	27 (28)
Outdoors	33(34)	33(34)
**Primary cooking fuel exposure**		
Wood	83 (86)	84 (87)
Agricultural residue	10 (10)	10(10)
Gas	3(3)	3(3)

^a^ Data are numbers (%) unless specifically indicated as mean (SD).

^b^ Five participants in the 12-year-old cohort were 11 years old and 12 participants in the 16-year-old cohort were 15 years old.

**Fig 1:**
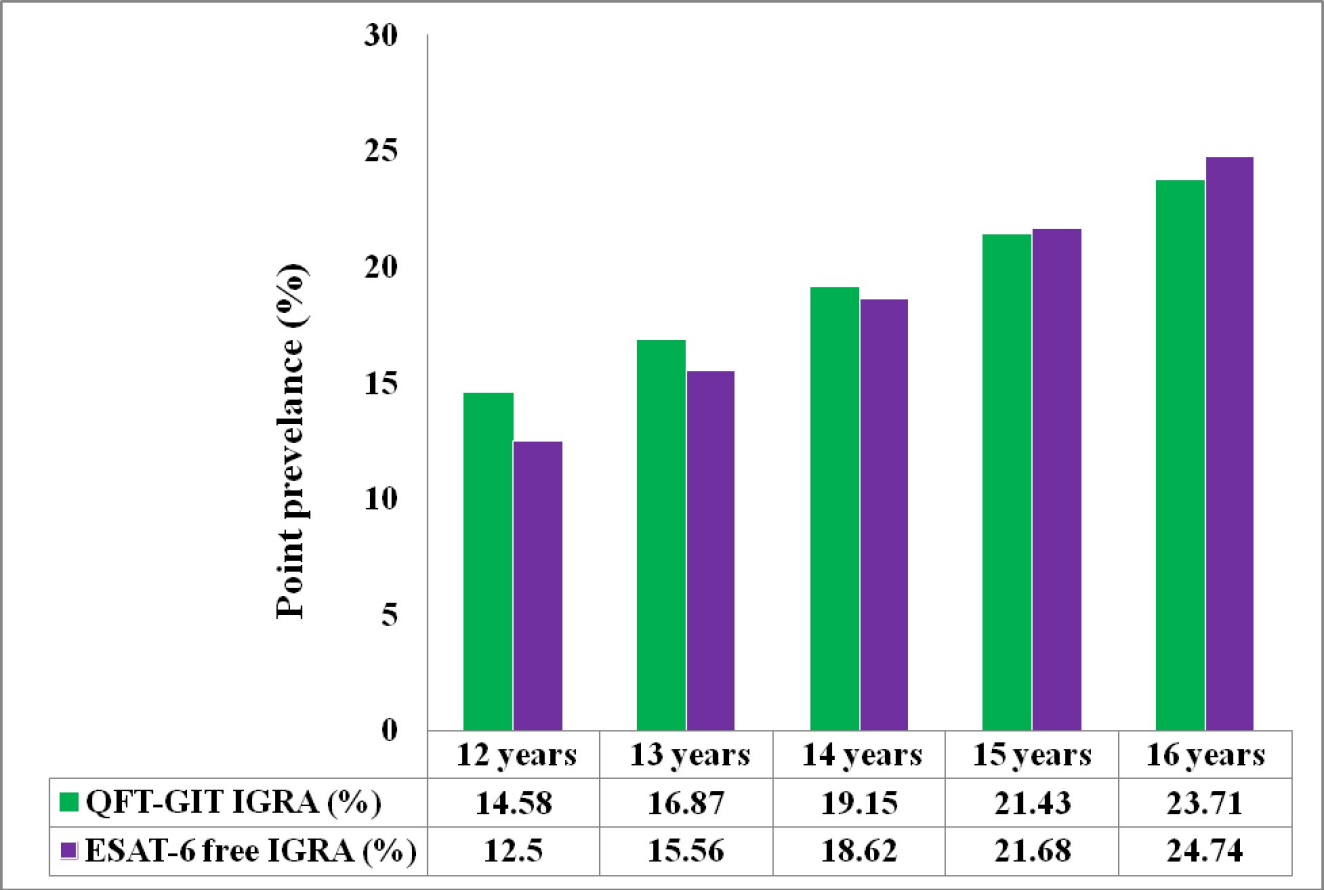
Bar chart summarizing estimates of latent tuberculosis infection (percentages) among adolescents by age for both the QFT-GIT IGRA and the novel ESAT-6 free IGRA tests. For 13—to 15-year-olds, the percentage data are estimates assuming a linear trend between prevalence determined in 12- and 16-year-olds.

Overall estimates of LTBI prevalence were 19.2% [95%CI, 14.1; 25.2] and 18.6% [95%CI, 13.6; 24.6] as measured by QFT-GIT IGRA and ESAT-6 Free IGRA, respectively. The 16-years-old cohort had a higher LTBI prevalence (23.7% [95%CI, 16.1; 32.9]) than the 12-year-old cohort (14.6% [95%CI, 8.6; 22.7]) as measured by QFT-GIT IGRA; a similar trend was observed as measured by ESAT-6 Free IGRA, with 24.7% [95%CI, 16.9; 34.0] among the 16-years-olds compared to 12.5% [95%CI, 7.0; 20.3] among the 12-year-olds. The LTBI prevalence by gender across both cohorts is summarised in Table 2, and the estimation of LTBI prevalence for ages 13, 14, and 15 is presented in Fig 1.

**Table 2:** Latent tuberculosis infection prevalence among 193 adolescents measured by QFT-GIT IGRA and ESAT-6 Free IGRA^a^.

	12 years old (n = 96)			16 years old (n = 97)		
	Positive cases^b^ (n)	Case tested^c^ (n)	Point prevalence (95% CI)^d^	Positive cases^b^ (n)	Case tested^c^ (n)	Point prevalence (95% CI)^d^
**QFT-GIT IGRA**						
Overall	14	96	14.58 (8.55; 22.73)	23	97	23.71 (16.05; 32.92)
Female	4	60	6.67 (2.15; 15.30)	14	50	28.00 (16.91; 41.58)
Male	10	36	27.78 (15.06; 43.95)	9	47	19.15 (9.76; 32.24)
**ESAT -6 free IGRA**						
Overall	12	96	12.50 (6.95; 20.28)	24	97	24.74 (16.93; 34.04)
Female	2	60	3.33 (0.56; 10.58)	14	50	28.00(16.91; 41.58)
Male	10	36	27.78 (15.06; 43.95)	10	47	21.28 (11.34; 34.66)

^a.^ Five participants in the 12-year-old cohort were 11 years old and 12 participants in the 16-year- old cohort were 15 years old

^b^ Positivity as measured by QFT-GIT IGRA and by ESAT-6 free IGRA, Indeterminate results are considered negative.

^c^ Includes all enrolled participants with available QFT-GIT IGRA and ESAT-6 free IGRA.

^d^ Point prevalence = (number of positive cases / total number tested)*100. 95% mid-point confidence intervals are shown.

The ARTI among participants as measured by QFT-GIT IGRA was 1.25% in the 12-year-old cohort and 1.63% in the 16 year old cohort. For the ESAT-6 Free IGRA it was 1.06% in the 12 year old cohort and 1.71% in the 16 year old cohort Table 3. The extrapolated ARTI for 13-, 14-, and 15 year olds was 2.7%, 2.8%, and 2.9% respectively for QFT-GIT IGRA. For the ESAT-6 Free IGRA the estimates were 3.6%, 3.7%, and 3.9% for 13-, 14-, and 15 year olds respectively.

**Table 3:** Annual risk of tuberculosis infection as measured by QFT-GIT IGRA and ESAT-6 Free IGRA.

	12 years old	16 years old
	ARTI^b^	ARTI^b^
**QFT-GIT IGRA**^a^		
All	0.013	0.016
Female	0.006	0.019
Male	0.026	0.016
**ESAT -6 free IGRA**^a^		
All	0.011	0.017
Female	0.003	0.019
Male	0.026	0.014

^a^ Calculations based on all 12- and 16-years-old participants with available QFT-GIT IGRA and ESAT- 6 free IGRA data.

^b^ ARTI is an age-specific measure of average risk of *M*.*tb* infection over the lifetime of the participant. ARTI = 1 –(1-P)^1/A^, where P is *M*.*tb*. prevalence of the age group and A is the mean age of the participants.

**Fig 2:**
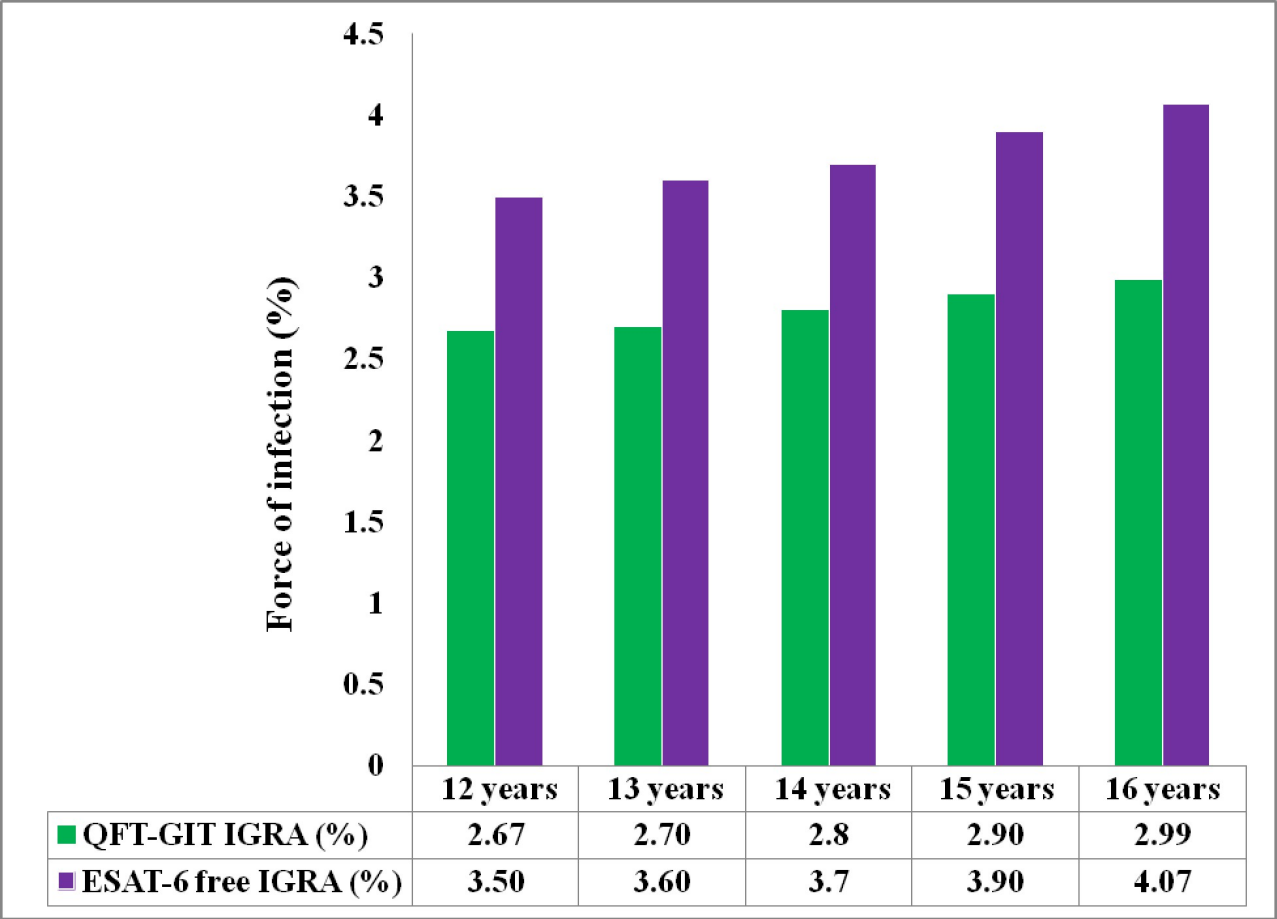
Bar chart summarizing estimates of tuberculosis force of infection (percentages) among adolescence for both the QFT-GIT IGRA and the novel ESAT-6 free IGRA tests. For 13- to 15-years-olds percentage data are estimates assuming a linear trend between force of infection determined in 12- and 16-years-olds.

Among all enrolled participants, the force of infection was 2.7% [95%CI, 0; 6.2] in the 12 year old cohort and 3.0% [95%CI, 0; 6.9] in the 16-year-old cohort by QFT-GIT IGRA, and it was 3.5% [95%CI, 0; 7.5] in the 12-year-old cohort and 4.7% [95%CI, 0; 8.7] in the 16-year-old cohort by ESAT-6 Free IGRA. The estimation of force of infection at ages 13, 14, and 15 is shown in Fig 2. Of all enrolled participants, 97.4% had concordant results for QFT-GIT IGRA and ESAT-6 Free IGRA (p = 0.65) (Table 4).

**Table 4:** Concordance between QFT-GIT and ESAT-6 free IGRA^a^.

ESAT—6 free IGRA	QFT-GIT IGRA		
	Positive, n (%)	Negative, n (%)	P-value
Positive, n (%)	34 (91.9)	2 (1.3)	0.65
Negative, n (%)	3 (8.1)	154 (98.7)	

^a^ Concordance based on all available IGRA data. Indeterminate results are regarded as negative. Positivity thresholds: QFT-GIT ≥ 0.35 IU/mL, ESAT-6F ≥ 0.61 IU/mL.

P-value is based on McNemar’s test; Kappa Coefficient: K = 0.92

## Discussion

We conducted an epidemiological study in Mwanza, Tanzania to estimate the rate of *M*.*tb*.infection to facilitate the design of POI studies in this region. We evaluated the prevalence of LTBI using a commercially available IGRA, QFT-GIT, as its utility in settings with a high TB burden and a high risk of infection has been demonstrated. [12, 13]. We compared the performance of QFT-GIT to the ESAT-6 free IGRA assay to explore the force of infection, ARTI, and prevalence of LTBI.

We found that there is a high prevalence of LTBI and corresponding ARTI suggesting ongoing transmission in this population. There was a high concordance between the QFT-GIT and the novel ESAT-6 free IGRA assays in the estimates of *M*.*tb*. infection which was similar both to a study by Ruhwald M. *et al*., where the agreement between ESAT-6 free IGRA using IFN-γ and QFT was high (~80%) [21], and to a study by Nemes E. *et al*., who reported a significant correlation with 91% concordance between the tests [23]. The concordance of the ESAT-6 free IGRA [21] with QFT suggests it might be an alternative to the regular IFN-γ release assay in TB vaccine trials where antigens in the vaccine are similar to those in the QFT assay. Although IGRA assays are the currently accepted tests to assess the efficacy of TB vaccine candidates and to estimate LTBI, the diagnosis of LTBI remains challenging, as there is no gold standard for LTBI determination [26, 27]. The high incidence of *M*.*tb*. infection in this population, as estimated by both tests, was observed to increase with age. These findings are similar to those reported in the age and sex model study done in Zambian and South African communities [28] and by Nduba V. *et al*. in western Kenya [29]. Our findings, plus those reported elsewhere [10–13, 30, 31], indicate that *M*.*tb*. transmission is more intense among adolescents than among young children. The high ARTI and corresponding force of infection observed by age in this study also confirm this trend. In this study, the ARTI, an epidemiological index that measures the extent of *M*.*tb*. transmission in the community was observed to range from 1.3% to 2.9%, similar to the force of TB infection estimates (proportion of susceptible individuals that have become infected with *M*.*tb*. in specified period) that ranges from 2.6% to 3.0%. The observed ARTI and force-of-infection estimates support a higher transmission of *M*.*tb*. infection in this region than was reported before [32–34] and are consistent with findings in South Africa, where an ARTI of 2%–4% was reported among adolescents [35].

A high prevalence of LTBI among adolescents in this population was not a surprise. Our previous study among adults (18 years old and above) within the same community population reported a high rate of LTBI among household contacts of TB patients as compared to neighbourhood controls [18]. The history of TB contact, the mixed socio-economic markers, and high social density in this population can explain the high rate of *M*.*tb*. infection observed.

Given that the prevalence of LTBI among adolescents in Mwanza region was unknown, despite the fact that the region reports the highest rate of TB disease and number of TB case notifications yearly [17], our findings provide good estimates of force of infection. Such results will help local and international investigators planning to conduct POI studies within the region. Our findings will both help to accurately determine the sample size of such trials and assist the TB control programmes in measuring the effectiveness of the programme implemented. In addition, these data suggest that a vaccine targeting adolescents and young adults could have a role in preventing *M*.*tb*. infection and thus avoid latent infection before they reach adulthood.

Although the study has limited numbers it does provide a valuable method to determine prevalence and estimated ARTI and force of infection in other regions or countries.

## Conclusions

We assessed the prevalence and ARTI among adolescents aged 12 and 16 years using two different IGRAs, the QFT-GIT and a novel ESAT-6 free assays, and found a high prevalence of LTBI and estimates of ARTI and force of infection. There was a clear indication that adolescents are a relevant population to assess the effect of new TB vaccine candidates targeting uninfected individuals as our results indicate ongoing transmission between 12 and 16 years of age. This data indicates that adolescents should be considered in global TB vaccine research efforts. Moreover, QFT-GIT and ESAT-6 free assays were reasonably similar in estimating LTBI indicating that the ESAT-6 free assay may be a good alternative if an investigational vaccine contains antigens presented in the QFT-GIT assay.

## References

[1] Global tuberculosis report 2020. Geneva: World Health Organization; 2020. Licence: CC BY-NC-SA 3.0 IGO

[2] The Stop TB Partnership. 12 months of COVID-19 eliminated 12 years of progress in the global fight against tuberculosis. March 18, 2021. http://www.stoptb.org/news/stories/2021/ns21_011.html.

[3] Getahun H MA, Chaisson RE, Raviglione M. (2015) Latent Mycobacterium tuberculosis infection. N Engl J Med 372: 2127–2135. doi: 10.1056/NEJMra1405427 26017823

[4] Fan WC, Ting WY, Lee MC, Huang SF, Chiu CH, et al. (2014) Latent TB infection in newly diagnosed lung cancer patients—A multicenter prospective observational study. Lung Cancer 85: 472–478. doi: 10.1016/j.lungcan.2014.07.001 25063540

[5] Evans TG BM, Barker L, Thole J (2013) Preventive vaccines for tuberculosis. Vaccine 18: B223–226. doi: 10.1016/j.vaccine.2012.11.081 23598486

[6] Khoshnood S, Heidary M, Haeili M, Drancourt M, Darban-Sarokhalil D, et al. (2018) Novel vaccine candidates against Mycobacterium tuberculosis. Int J Biol Macromol 120: 180–188. doi: 10.1016/j.ijbiomac.2018.08.037 30098365

[7] Dye C WB (2000) Tuberculosis 2000–2010: control, but not elimination. Int J Tuberc Lung Dis 4: S146–152. 11144545

[8] Dye C WB (2008) Eliminating human tuberculosis in the twenty-first century. J R Soc Interface 2008 5: 653–662. doi: 10.1098/rsif.2007.1138 17690054 PMC3226985

[9] Abu-Raddad LJ SL, Achterberg JT, Sugimoto JD, Longini IM Jr, Dye C, Halloran ME. (2009) Epidemiological benefits of more-effective tuberculosis vaccines, drugs, and diagnostics. Proc Natl Acad Sci U S A 106: 13980–13985. doi: 10.1073/pnas.0901720106 19666590 PMC2720405

[10] Wood R, Liang H, Wu H, Middelkoop K, Oni T, et al. (2010) Changing prevalence of tuberculosis infection with increasing age in high-burden townships in South Africa. Int J Tuberc Lung Dis 14: 406–412. 20202297 PMC2837545

[11] Mahomed H, Ehrlich R, Hawkridge T, Hatherill M, Geiter L, et al. (2013) TB incidence in an adolescent cohort in South Africa. PLoS One 8: e59652. doi: 10.1371/journal.pone.0059652 23533639 PMC3606161

[12] Mahomed H, Hawkridge T, Verver S, Geiter L, Hatherill M, et al. (2011) Predictive factors for latent tuberculosis infection among adolescents in a high-burden area in South Africa. Int J Tuberc Lung Dis 15: 331–336. 21333099

[13] Mahomed H, Hawkridge T, Verver S, Abrahams D, Geiter L, et al. (2011) The tuberculin skin test versus QuantiFERON TB Gold(R) in predicting tuberculosis disease in an adolescent cohort study in South Africa. PLoS One 6: e17984. doi: 10.1371/journal.pone.0017984 21479236 PMC3066222

[14] Machingaidze S VS, Mulenga H, Abrahams DA, Hatherill M, Hanekom W, Hussey GD, et al. (2012) Predictive value of recent QuantiFERON conversion for tuberculosis disease in adolescents. Am J Respir Crit Care Med 186: 1051–1056. doi: 10.1164/rccm.201206-1134OC 22955316

[15] Ellis RD, Hatherill M, Tait D, Snowden M, Churchyard G, et al. (2015) Innovative clinical trial designs to rationalize TB vaccine development. Tuberculosis (Edinb) 95: 352–357. doi: 10.1016/j.tube.2015.02.036 25802031

[16] Nemes E, Geldenhuys H, Rozot V, Rutkowski KT, Ratangee F, et al. (2018) Prevention of M. tuberculosis Infection with H4:IC31 Vaccine or BCG Revaccination. N Engl J Med 379: 138–149. doi: 10.1056/NEJMoa1714021 29996082 PMC5937161

[17] The National Tuberculosis and leprosy Programme Annual report for 2017https://ntlp.go.tz/site/assets/files/1046/ntlp_annual_report_2017_final.pdf.

[18] Jensen AV JL, Faurholt-Jepsen D, Aabye MG, Praygod G, Kidola J, Faurholt-Jepsen M, et al. (2013) The prevalence of latent Mycobacterium tuberculosis infection based on an interferon-γ release assay: a cross-sectional survey among urban adults in Mwanza, Tanzania. PLoS One 8: e64008. doi: 10.1371/journal.pone.0064008 23700446 PMC3660306

[19] McNerney R MM, Abubakar I, Marais B, McHugh TD, Ford N, Weyer K, et al. (2012) Tuberculosis diagnostics and biomarkers: needs, challenges, recent advances, and opportunities. J Infect Dis 205 Suppl 2. 10.1093/infdis/jir860 22496353

[20] Luabeya AK, Kagina BM, Tameris MD, Geldenhuys H, Hoff ST, et al. (2015) First-in-human trial of the post-exposure tuberculosis vaccine H56:IC31 in Mycobacterium tuberculosis infected and non-infected healthy adults. Vaccine 33: 4130–4140. doi: 10.1016/j.vaccine.2015.06.051 26095509

[21] Ruhwald M, de Thurah L, Kuchaka D, Zaher MR, Salman AM, et al. (2017) Introducing the ESAT-6 free IGRA, a companion diagnostic for TB vaccines based on ESAT-6. Sci Rep 7: 45969. doi: 10.1038/srep45969 28387329 PMC5384086

[22] Statistics TNBo (2012) Data Portal. 2012 Census Data. Population Distribution by Age and Sex. Dar es Salaam.

[23] Nemes E, Abrahams D, Scriba TJ, Ratangee F, Keyser A, et al. (2019) Diagnostic Accuracy of Early Secretory Antigenic Target-6-Free Interferon-gamma Release Assay Compared to QuantiFERON-TB Gold In-tube. Clin Infect Dis 69: 1724–1730. doi: 10.1093/cid/ciz034 30668657 PMC6821223

[24] Cellestis (2015) QuantiFERON-TB Gold package insert. Cellestis, a QIAGEN Company, Chadstone, Victoria, Australia.

[25] Middelkoop K BL, Liang H, Aquino LD, Sebastian E, Myer L, Wood R. (2011) Force of tuberculosis infection among adolescents in a high HIV and TB prevalence community: a cross-sectional observation study. BMC Infect Dis 11: 156. doi: 10.1186/1471-2334-11-156 21631918 PMC3130671

[26] (2000) Targeted tuberculin testing and treatment of latent tuberculosis infection. This official statement of the American Thoracic Society was adopted by the ATS Board of Directors, July 1999. This is a Joint Statement of the American Thoracic Society (ATS) and the Centers for Disease Control and Prevention (CDC). This statement was endorsed by the Council of the Infectious Diseases Society of America. (IDSA), September 1999, and the sections of this statement. Am J Respir Crit Care Med 161: S221–247. doi: 10.1164/ajrccm.161.supplement_3.ats600 10764341

[27] Pai M, Denkinger CM, Kik SV, Rangaka MX, Zwerling A, et al. (2014) Gamma interferon release assays for detection of Mycobacterium tuberculosis infection. Clinical microbiology reviews 27: 3–20. doi: 10.1128/CMR.00034-13 24396134 PMC3910908

[28] Dodd PJ, Looker C, Plumb ID, Bond V, Schaap A, et al. (2016) Age- and Sex-Specific Social Contact Patterns and Incidence of Mycobacterium tuberculosis Infection. Am J Epidemiol 183: 156–166. doi: 10.1093/aje/kwv160 26646292 PMC4706676

[29] Nduba V, Van’t Hoog AH, de Bruijn A, Mitchell EMH, Laserson K, et al. (2019) Estimating the annual risk of infection with Mycobacterium tuberculosis among adolescents in Western Kenya in preparation for TB vaccine trials. BMC Infect Dis 19: 682. doi: 10.1186/s12879-019-4314-7 31375068 PMC6679456

[30] Kifai EJ, Bakari M (2009) Mantoux skin test reactivity among household contacts of HIV-infected and HIV un-infected patients with sputum smear positive TB in Dar es Salaam, Tanzania. East Afr J Public Health 6: 211–218. doi: 10.4314/eajph.v6i2.51786 20000032

[31] Triasih R, Robertson C, Duke T, Graham SM (2015) Risk of infection and disease with Mycobacterium tuberculosis among children identified through prospective community-based contact screening in Indonesia. Trop Med Int Health 20: 737–743. doi: 10.1111/tmi.12484 25704441

[32] Egwaga SM, Cobelens FG, Muwinge H, Verhage C, Kalisvaart N, et al. (2006) The impact of the HIV epidemic on tuberculosis transmission in Tanzania. AIDS 20: 915–921. doi: 10.1097/01.aids.0000218557.44284.83 16549977

[33] Migliori GB, Borghesi A, Spanevello A, Eriki P, Raviglione M, et al. (1994) Risk of infection and estimated incidence of tuberculosis in northern Uganda. Eur Respir J 7: 946–953. 8050553

[34] Odhiambo JA, Borgdorff MW, Kiambih FM, Kibuga DK, Kwamanga DO, et al. (1999) Tuberculosis and the HIV epidemic: increasing annual risk of tuberculous infection in Kenya, 1986–1996. Am J Public Health 89: 1078–1082. doi: 10.2105/ajph.89.7.1078 10394319 PMC1508825

[35] Middelkoop K, Bekker L-G, Myer L, Dawson R, Wood R (2008) Rates of Tuberculosis Transmission to Children and Adolescents in a Community with a High Prevalence of HIV Infection among Adults. Clinical Infectious Diseases 47: 349–355. doi: 10.1086/589750 18558885 PMC3816246

